# RETRACTION: Negative Pressure Wound Therapy for Patients With Mediastinitis: A Meta‐Analysis

**DOI:** 10.1111/iwj.70646

**Published:** 2025-04-23

## Abstract

**RETRACTION:** WangZ. , FengC. , and WangX.‐J. , “Negative Pressure Wound Therapy for Patients With Mediastinitis: A Meta‐Analysis,” International Wound Journal 17, no. 6 (2020): 2019–2025, 10.1111/iwj.13494.32856392 PMC7949316

The above article, published online on 27 August 2020, in Wiley Online Library (http://onlinelibrary.wiley.com/), has been retracted by agreement between the journal Editor in Chief, Professor Keith Harding; and John Wiley & Sons Ltd. Following an investigation by the publisher, all parties have concluded that this article was accepted solely on the basis of a compromised peer review process. The editors have therefore decided to retract the article. The corresponding author could not be reached for comment regarding the retraction.

**RETRACTION:** Z. Wang , C. Feng , and X.‐J. Wang , “Negative Pressure Wound Therapy for Patients With Mediastinitis: A Meta‐Analysis,” International Wound Journal 17, no. 6 (2020): 2019–2025, 10.1111/iwj.13494.32856392 PMC7949316

The above article, published online on 27 August 2020, in Wiley Online Library (http://onlinelibrary.wiley.com/), has been retracted by agreement between the journal Editor in Chief, Professor Keith Harding; and John Wiley & Sons Ltd. Following an investigation by the publisher, all parties have concluded that this article was accepted solely on the basis of a compromised peer review process. The editors have therefore decided to retract the article. The corresponding author could not be reached for comment regarding the retraction.

